# A Hybrid Method Based on Extreme Learning Machine and Self Organizing Map for Pattern Classification

**DOI:** 10.1155/2020/2918276

**Published:** 2020-08-25

**Authors:** Imen Jammoussi, Mounir Ben Nasr

**Affiliations:** Control and Energy Management Laboratory (CEMLab), Department of Electrical Engineering, ENIS, Sfax 1173, Tunisia

## Abstract

Extreme learning machine is a fast learning algorithm for single hidden layer feedforward neural network. However, an improper number of hidden neurons and random parameters have a great effect on the performance of the extreme learning machine. In order to select a suitable number of hidden neurons, this paper proposes a novel hybrid learning based on a two-step process. First, the parameters of hidden layer are adjusted by a self-organized learning algorithm. Next, the weights matrix of the output layer is determined using the Moore–Penrose inverse method. Nine classification datasets are considered to demonstrate the efficiency of the proposed approach compared with original extreme learning machine, Tikhonov regularization optimally pruned extreme learning machine, and backpropagation algorithms. The results show that the proposed method is fast and produces better accuracy and generalization performances.

## 1. Introduction

The extreme learning machine (ELM) is a very important supervised machine learning algorithm proposed for training single hidden layer feedforward neural network (SLFN), which have been successfully used in many engineering disciplines [1–8], etc. One of the main drawbacks of ELM is the selection of the optimal number of hidden nodes, the random choose of the input parameters, and the type of the activation functions. These disadvantages directly affect the performances of neural network [9, 10]. Therefore, in order to enhance the performance of SLFN, several algorithms have been developed for optimizing ELM hidden nodes [11–23]. In [11], the authors proposed a new kind of ELM, named self-adaptive extreme learning machine (SaELM), in which optimal hidden neurons number are selected to construct the neural network. In [12], Huang et al. proposed an incremental extreme learning machine, named (I-ELM), which randomly adds hidden neurons incrementally and analytically determines the output weights. In [13], Huang and Chen proposed an improved version for (I-ELM) called enhanced random search-based incremental algorithm (EI-ELM), which choose the hidden neurons that lead to the smallest residual error at each learning step. A further improvement about (I-ELM) is made in convex incremental extreme learning machine (CI-ELM) [14]. Its output weights are updated after a new hidden neuron is added. In [15], an effective learning algorithm, known as self-adaptive evolutionary extreme learning machine, is presented to adjust the ELM input parameters adaptively, which improves the generalization performance of ELM. An improved evolutionary extreme learning machine based on particle swarm optimization was proposed to find the optimal input weights and hidden biases [16]. Error minimized extreme learning machine (EM-ELM) [17] randomly adds neurons to the hidden layer one by one or group by group and updates output weights recursively. Pruned-ELM [18], named as P-ELM, was presented to determine the number of hidden neurons using statistical methods. In [19], Miche et al. considered the optimally pruned extreme learning machine (OP-ELM), in which the hidden neurons are ranked using multiresponse sparse regression algorithm, and then the selection for the best number of neurons is taken by a leave-one-out validation method. In [20], a constructive hidden neuron selection ELM (CS-ELM) was proposed, where the hidden neurons are selected according to some criteria. The work in [21] used ELM with adaptive growth of hidden neurons (AG-ELM) to automate the design of networks. In [22], by combining Bayesian models and ELM, the Bayesian ELM (BELM) is proposed to optimize the weights of the output layer using probability distribution. In [23], Miche et al. proposed a double regularized ELM using a least-angle regression (LARS) and Tikhonov regularization (TROP-ELM). Bidirectional extreme learning machine (B-ELM) was presented in [24], in which some hidden neurons are not randomly selected. In [25], Cao et al. proposed an enhanced bidirectional extreme learning machine (EB-ELM), in which some hidden neurons are randomly generated and only the neurons with the largest residual error are added to the existing network. Online sequential learning mode based on ELM (OS-ELM) was presented in [26]. Fuzziness based OS-ELM was presented in [27]. In [28], a dynamic forgetting factor is utilised to adjust OS-ELM parameters, and the corresponding DOS-ELM algorithm is proposed. Up to now, many other algorithms have been considered to extend the basic ELM to make it more efficient [29–35].

Motivated by developing a fast and efficient training algorithm for SLFN, this paper presents a new hybrid approach for training SLFN, where the weights between the input layer and the hidden layer are optimized by a self-organizing map algorithm [36], and the output weights are calculated using the Moore–Penrose generalized inverse like in ELM [1]. The efficiency in terms of classification accuracy and computation time of the proposed method is shown by the simulation results of different classification problems. The main contributions of our work can be summarized as follows:

1. We propose a hybrid algorithm combining the self-organizing map algorithm with extreme learning machine algorithm for optimizing SLFN weights. In this algorithm, the self-organizing map is first used to optimize the weights connecting the input and hidden layers. Then, the ELM is applied to determine the weights connecting the hidden and output layers. The main objective of the proposed approach is to achieve a higher solution accuracy and faster convergence with a compact network size.
2. Comparing with various methods, we evaluate the performance of our algorithm in terms of classification accuracy and convergence speed over different types of datasets.

The remainder of this paper is as follows. In Section 2, we recall the preliminary of ELM.  Section 3 provides a detailed description of the hybrid learning algorithm. In Section 4, simulation results and comparisons with BP algorithm, basic ELM, and TROP-ELM are given. Finally, the conclusion is drawn in Section 5.

## 2. Basic ELM Algorithm

**Figure 1:**
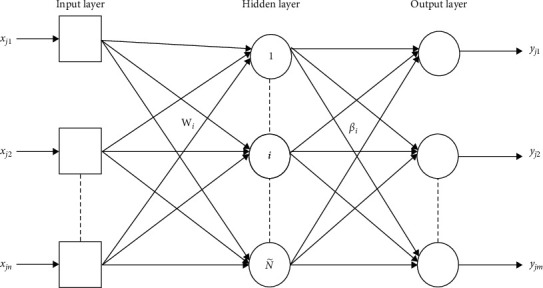
Single hidden layer feedforward neural network (SLFN).

Recently, an efficient learning algorithm, called extreme learning machine (ELM), for single hidden layer feedforward neural network (SLFN) has been proposed by Huang et al. [1]. In ELM, the input weights of the hidden nodes are randomly chosen, and the output weights of SLFN are then computed by using the pseudoinverse operation of the hidden layer output matrix. The illustration of single hidden layer feedforward neural network is given in Figure 1. The numbers of neurons for input, hidden, and output layers are *n*, $\tilde{N\;}$, and *m*, respectively.

Given *N* training samples (**x**_*j*_, **t**_*j*_), where **x**_*j*_= [*x*_*j*1_, *x*_*j*2_,…,*x*_*jn*_]^T^ ∈ *ℝ*^*n*^ and **t**_*j*_=[*t*_*j*1_, *t*_*j*2_,…,*t*_*jm*_]^T^ ∈ *ℝ*^*m*^. The output of an SLFN can be represented by:

(1) $$\begin{array}{c} \;\overset{\tilde{N}}{\underset{i=1}{\sum }}\beta_{i\;}f\left(w_{i}.x_{j}+b_{i}\right)=y_{j},\;j=1\ldots N, \end{array}$$

where **w**_*i*_= [*w*_*i*1_, *w*_*i*2_,…,*w*_*in*_]^T^ is the weight vector connecting the *i*^*th*^ hidden node and the input nodes.

In general, the total weight matrix W is

(2) $$\begin{array}{c} \;W_{\tilde{N}\times n}=\left[w_{1};w_{2};\ldots ;w_{\tilde{N}}\right]={\left(\begin{array}{cccc} w_{11} & w_{12} & \cdots & w_{1n}\\ w_{21} & w_{22} & \cdots & w_{2n}\\ \vdots & \vdots & \ddots & \vdots \\ w_{\tilde{N}1} & w_{\tilde{N}2} & \cdots & w_{\tilde{N}n} \end{array}\right)}_{\tilde{N}\times n}. \end{array}$$

where **β**_**i**_= [*β*_*i*1_, *β*_*i*2_,…,*β*_im_]^T^  is the weight vector connecting the *i*^th^ hidden node and the output nodes, *b*_*i*_ is the threshold of the *i*^th^ node, **y**_*j*_= [*y*_*j*1_, *y*_*j*2_, ⋯,*y*_*jm*_]^T^ ∈ *ℝ*^*m*^ is the output vector of neural network, and *f*(.)  denotes an activation function, in general, *f*(*x*)= 1/(1+*e*^−*x*^) 

Equation (1) can be written compactly as

(3) $$\begin{array}{c} H\beta =Y, \end{array}$$

where *H* is the output matrix of the hidden layer and defined as follows:

(4) $$\begin{array}{c} H\left(w_{1},\ldots ,w_{\tilde{N}},b_{1},\ldots ,b_{\tilde{N}},x_{1},\ldots ,x_{N}\;\right)={\left(\begin{array}{ccc} \cdots & f\left(w_{\tilde{N}}.x_{1}+b_{\tilde{N}}\right)\\ \vdots & \cdots & \vdots \\ \vdots & \cdots & \vdots \\ f\left(w_{1}.x_{N}+b_{1}\right) & \cdots & f\left(w_{\tilde{N}}.x_{N}+b_{\tilde{N}}\right) \end{array}\right)}_{N\times \tilde{N}}, \end{array}$$

(5) $$\begin{array}{c} \beta =\;{\left(\begin{array}{c} \vdots \\ \beta_{\tilde{N}}^{T} \end{array}\right)}_{\tilde{N}\times m}\;, \end{array}$$

The criterion function to be minimized is the sum of the squared errors over all the training samples, given by

(6) $$\begin{array}{c} E={\left\|Y-T\right\|}^{2}=\;{\left\|H\beta -T\right\|}^{2}. \end{array}$$

The output weight matrix can be determined analytically by minimizing the least square error:

(7) $$\begin{array}{c} \hat{\beta }=\arg\underset{\beta }{\min}{\left\|H\beta -T\right\|}^{2}. \end{array}$$

A solution of the linear system (7), *β*, can be computed as follows:

(8) $$\begin{array}{c} \hat{\beta }=H^{+}T\;, \end{array}$$

where *H*^+^  is called the Moore–Penrose generalized inverse of matrix *H* and *T* is the desired output matrix, expressed as

(9) $$\begin{array}{c} \;T=\;{\left(\begin{array}{c} t_{1}^{T}\\ \vdots \\ t_{N}^{T} \end{array}\right)}_{N\times m}. \end{array}$$

The ELM algorithm can be summarized as follows:

Step 1. Randomly assign the input weight **w**_i_ and biases *b*_*i*_, *i* ∈ [1,$\;\tilde{N}$].
Step 2. Calculate the hidden layer output matrix *H* using equation (4).
Step 3. Calculate the output weight matrix by equation (8).

## 3. Proposed Learning Algorithm

**Figure 2:**
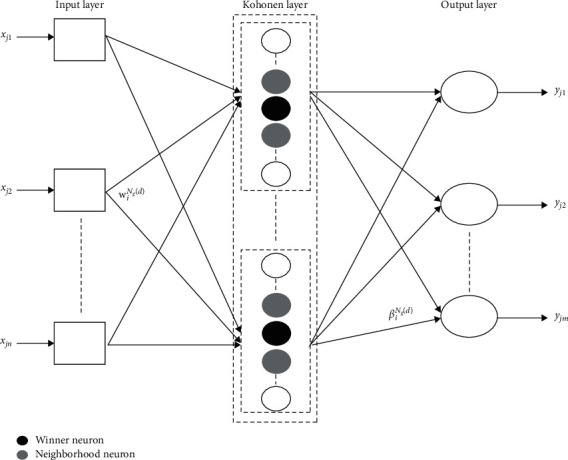
SLFN structure with one-dimensional Kohonen layer.

In this study, the architecture of the proposed single hidden layer feedforward neural network (SLFN) is shown in Figure 2.

**Figure 3:**
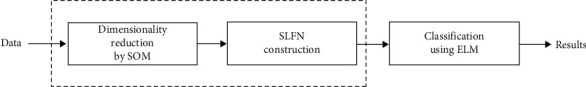
The sketch map of the proposed method.

It is composed of an input layer, one-dimensional Kohonen layer, and an output layer. To ensure the superiority of the proposed network structure, an appropriate hybrid learning algorithm for training a SLFN is presented. This algorithm is the fusion of a self-organizing map [36] and extreme learning machine [1]. During training with this algorithm, the network operates in a two-stage sequence. The weights of hidden layer are clustered by SOM in the first stage. In the second stage, ELM is initialized with the weights obtained in the previous stage. The sketch map of the proposed method is shown in Figure 3.

The learning algorithm can be described as follows.

### 3.1. Stage 1: SOM-Based Initialization

Self-organizing map (SOM) is an unsupervised learning method to represent high-dimensional data vectors into a regular low-dimensional map by grouping similar input vectors and form a number of clusters. In our work, the basic SOM network consists of two layers, an input layer and a one-dimensional Kohonen layer in which neurons are arranged into a one-dimensional map. Each neuron *i* on the map is presented by *n*-dimensional weight vector **w**_i_=[*w*_*i*1_, *w*_*i*2_, ⋯,*w*_*in*_]^*T*^, where *n* is the dimension of the input vector **x**. The steps of SOM learning algorithm are as follows:

Step 1. Initialize weights to small random values, and initialize the neighborhood size.
Step 2. Select a vector **x**_*j*_ and determine the index of the winner neuron *g*, that is,
  (10) $$\begin{array}{c} \text{g}\left(x_{\text{j}}\right)=\text{arg}\underset{i}{\min}\left\|x_{\text{j}}-w_{\text{i}}\right\|,\;i=1,\ldots ,\tilde{N}, \end{array}$$
  where $\tilde{N}$ is the total number of neurons in the Kohonen layer.
Step 3. Update the weight of the winning neuron and its neighbor using the following Kohonen rule.
  (11) $$\begin{array}{c} w_{i}^{N_{g}\left(d\right)}\left(t+1\right)=w_{i}^{N_{g}\left(d\right)}\left(t\right)+\alpha \left(x_{j}\left(t\right)-w_{i}^{N_{g}\left(d\right)}\left(t\right)\right),\;i\;\in \;N_{g}\left(d\right), \end{array}$$
  where the neighborhood *N*_*g*_(*d*)  contains the indices for all of the neurons that lie within a radius *d* of the winning neuron *g* and *α*  is the learning rate.
Step 4. If all input data **x**_*j*_  are presented to the network, go to Step 5; otherwise, go to Step 2.

### 3.2. Stage 2: ELM with Subset of Neurons

In the first stage, SOM is used to reduce the dimension of input weights matrix *W* of ELM from $\tilde{N}\times n$ to $\tilde{n}\times n$.

Step 5. Create a weight matrix from input layer to the Kohonen layer and insert the values of each weight in the matrix as follows:
  (12) $$\begin{array}{c} W_{\tilde{n}\times n}^{N_{g}\left(d\right)}=\left[w_{1}^{N_{g}\left(d\right)};w_{2}^{N_{g}\left(d\right)};\ldots ;w_{\tilde{n}}^{N_{g}\left(d\right)}\right]={\left(\begin{array}{cccc} w_{11}^{N_{g}\left(d\right)} & w_{12}^{N_{g}\left(d\right)} & \cdots & w_{1n}^{N_{g}\left(d\right)}\\ w_{21}^{N_{g}\left(d\right)} & w_{22}^{N_{g}\left(d\right)} & \cdots & w_{2n}^{N_{g}\left(d\right)}\\ \vdots & \vdots & \ddots & \vdots \\ w_{\tilde{n}1}^{N_{g}\left(d\right)} & w_{\tilde{n}2}^{N_{g}\left(d\right)} & \cdots & w_{\tilde{n}n}^{N_{g}\left(d\right)} \end{array}\right)}_{\tilde{n}\times n}, \end{array}$$
  where **w**_*r*_^*N*_*g*_(*d*)^ are the weights of the winner neuron and its neighbors in Kohonen layer, $r\;\in \left\{1,2,\ldots ,\;\tilde{n}\right\}\;$ represents the order of the corresponding weight vector, and $\tilde{\text{n}}$ is the number of all neurons in the set *N*_*g*_.
Step 6. Set the final $W_{\tilde{n}\times n}^{N_{g}\left(d\right)}$ as initial weight matrix of the ELM.
Step 7. Calculate the hidden layer output matrix *H*^*N*_*g*_^ for input **x**:
  (13) $$\begin{array}{c} H^{N_{g}}\left(w_{1}^{N_{g}\left(d\right)},\ldots ,w_{\tilde{n}}^{N_{g}\left(d\right)},b_{1}^{N_{g}\left(d\right)},\ldots b_{\tilde{n}}^{N_{g}\left(d\right)},x_{1},\ldots ,x_{N}\;\right)={\left(\begin{array}{ccc} f\left(w_{1}^{N_{g}\left(d\right)}.x_{1}+b_{1}^{N_{g}\left(d\right)}\right) & \cdots & f\left(w_{\tilde{n}}^{N_{g}\left(d\right)}.x_{1}+b_{\tilde{n}}^{N_{g}\left(d\right)}\right)\\ \vdots & \cdots & \vdots \\ \vdots & \cdots & \vdots \\ f\left(w_{1}^{N_{g}\left(d\right)}.x_{N}+b_{1}^{N_{g}\left(d\right)}\right) & \cdots & f\left(w_{\tilde{n}}^{N_{g}\left(d\right)}.x_{N}+b_{\tilde{n}}^{N_{g}\left(d\right)}\right) \end{array}\right)}_{N\times \tilde{n}}. \end{array}$$
Step 8. Calculate the weights between the hidden layer and the output layer:
  (14) $$\begin{array}{c} {\hat{\beta }}^{N_{g}\left(d\right)}=\;H^{N_{g}+}T, \end{array}$$
  where **β**_**i**_^*N*_*g*_(*d*)^= [*β*_*i*1_^*N*_*g*_(*d*)^, *β*_*i*2_^*N*_*g*_(*d*)^,…,*β*_*im*_^*N*_*g*_(*d*)^]^*T*^ is the new weight vector connecting the *i*^*th*^  hidden node and the output layer.

## 4. Simulation Results

In this section, simulation results are presented and discussed in order to evaluate the performance of the proposed algorithm and to compare it with the conventional BP algorithm, basic ELM, and TROP-ELM through a classification problem. Our method has been tried on nine datasets; the first eight datasets are from the UCI Machine Learning Repository. The ninth dataset “Jaffe” is composed of images and provided by the Psychology Department in Kyushu University. The algorithms were tested on a computer with the Core-i5 processor, 8 GB RAM, 2.4 GHz CPU, MATLAB R2018a.

### 4.1. Datasets Description

**Figure 4:**
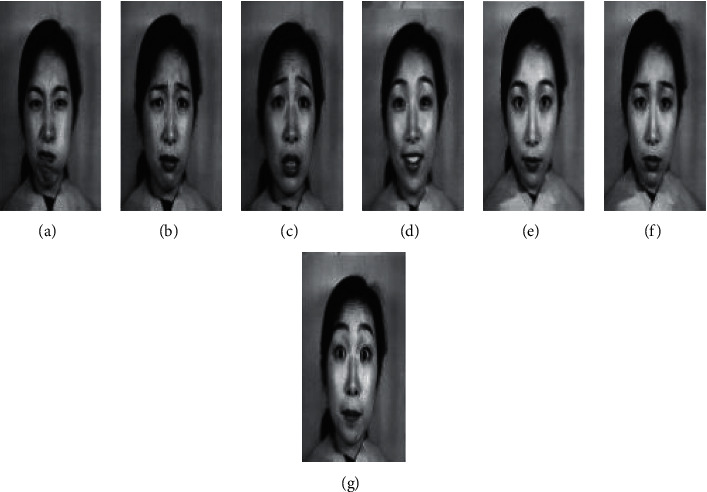
Samples of the Jaffe dataset. (a) Angry. (b) Disgust. (c) Fear. (d) Happy. (e) Neutral. (f) Sad. (g) Surprised.

There are many benchmarks for classifications, and we have selected nine classification datasets that are summarized in Table 1. The description of the datasets is as follows:

  Dataset 1: ionosphere is a type of dataset used for binary classification. The main objective is to determine the type of a given signal (good or bad) by referring to free electrons in the ionosphere. It has 351 instances divided into two classes with 34 integer and real attributes.
  Dataset 2: Iris is the most popular and the best-known dataset for classification and recognition of models based on the examination of the size of petals and sepals of the plant. It contains in totality 150 instances, which are equally separated between three classes. Each instance is characterized by four real attributes.
  Dataset 3: the wine dataset is the result of a chemical analysis of wines grown in the same region in Italy but derived from three different cultivars. It shows the existence of 178 instances and 13 continuous attributes.
  Dataset 4: the balance dataset is generated to model psychological experimental results. Four categorical attributes can indicate the balance scale of the 625 instances that are divided into three classes.
  Dataset 5: it is a simple dataset that consists of 101 animals from a Zoo. This dataset is able to predict the seven class of animals based on the 16 Boolean attributes.
  Dataset 6: this dataset includes 2310 instances divided into 7 classes that are handsegmented to create a classification for every pixel. Image data are described by 19 attributes.
  Dataset 7: the objective of the Ecoli dataset is to predict the localization of proteins by using measurements on the cell. It has 336 instances which are identified by seven attributes and divided into eight classes in unbalanced way.
  Dataset 8: the multiple features dataset aims to classify the handwritten numerals. It has in totality 2000 instances that are equally separated between 10 classes with 649 attributes.
  Dataset 9: the Jaffe dataset is composed of 213 grayscale images sized of 256∗256 and posed by 10 Japanese female models. Each female has two to four examples for each expression. The objective is to predict for each image one of the seven facial expressions such as angry, disgust, fear, happy, neutral, sad, and surprised. One emotion of the seven different facial expressions from the Jaffe dataset is shown in Figure 4.

For all datasets, 70% of the data are chosen for training phase while the remaining are reserved for testing. Three performance metrics have been listed in Table 2 in which accuracy value is calculated as follows:

(15) $$\begin{array}{c} \text{Accuracy}=\frac{\text{TP}+\text{TN}}{\text{TP}+\text{TN}+\text{FP}+\text{FN}}, \end{array}$$

where TP is the number of elements correctly classified as positive, FP is the number of positive elements incorrectly classified, FN is the number of negative elements incorrectly classified, and TN is the number of true elements correctly classified as negative.

### 4.2. Results and Discussion

**Figure 5:**
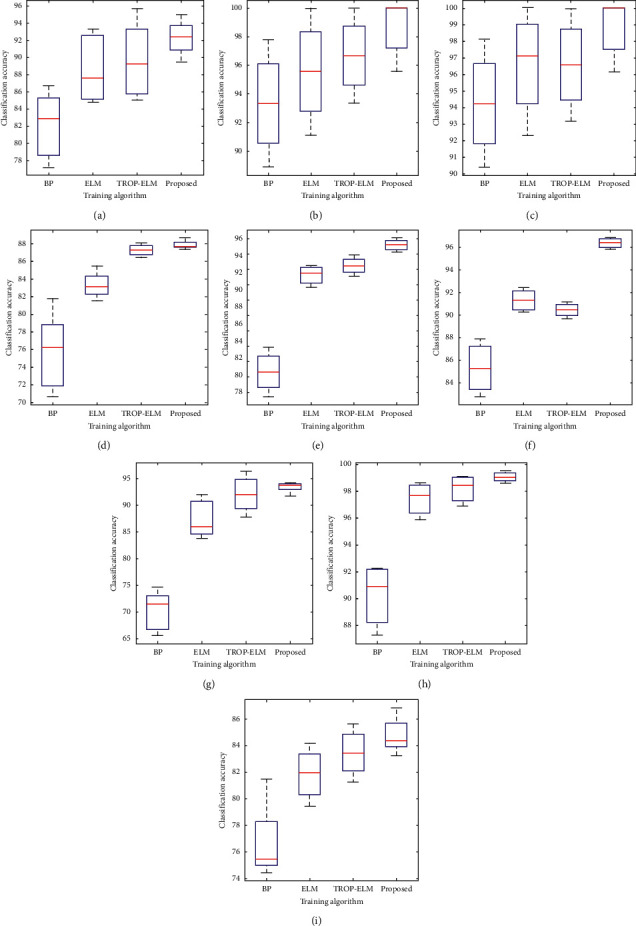
Box plots depicting the performance of training algorithms. Accuracy value variation of the (a) Ionosphere dataset, (b) Iris dataset, (c) Wine dataset, (d) Balance dataset, (e) Zoo dataset, (f) Image segmentation dataset, (g) Ecoli dataset, (h) Multiple features dataset, and (i) Jaffe dataset.

The performance of the current ELM method is dependent on the initial input weights and biases which are randomly initialized. In an attempt to overcome this problem, the heuristic approach explained above is used to automatically determine the optimal number of hidden neurons $\tilde{n}$ based on the clustering method. Different from basic ELM with $\tilde{N}$ hidden neurons, our method generally needs less hidden neurons and $\tilde{n}<\tilde{N}$. The comparison results given in Table 2 clearly indicate that our approach reduces the number of hidden neurons compared with the standard ELM and TROP-ELM for all cases. In addition, it should also be noted that the proposed approach outperforms the standard ELM, TROP-ELM, and backpropagation algorithms in terms of training time. A Box and Whiskers plot illustrations of the compared methods is shown in Figure 5. It can be clearly seen from Table 2 and Figure 5 that the accuracy of the results of the proposed algorithm is indeed higher than that of backpropagation, ELM, and TROP-ELM algorithms. All these results indicate that the hybrid algorithm can optimize the network structure to a suitable size with fewer hidden nodes and yet be able to classify the datasets with a better accuracy.

## 5. Conclusion

This paper proposed a novel hybrid algorithm for single hidden layer feedforward neural network. This algorithm consists of the use of a self-organizing map algorithm coupled with extreme learning machine. The learning process of this method includes two steps. The first step is to train the weights connecting the input and the hidden layers by a self-organizing map algorithm, and the second step is to use the Moore–Penrose inverse method to calculate the weights connecting the hidden and output layers. In order to prove the performance of the hybrid approach, it is used to solve several popular classification problems. A comparison with other basic methods such as BP, ELM, and TROP-ELM confirms the superiority of this method in terms of generalization performance and faster learning speed. The main disadvantage of the proposed method is that it uses a fixed structure of self-organizing map, where the number of neurons and the size of neighbourhood function must be determined before clustering. This often leads to significant limitation for most application. In future work, we will consider extending the study of the proposed method in the image classification domain. Another direction of future research includes the study of the proposed approach with different types of self-organizing maps and a wide range of activation functions.

## Figures and Tables

**Table 1:** Characteristics of the nine datasets.

Datasets	Training data	Testing data	Attributes	Classes
Ionosphere	246	105	34	2
Iris	105	45	4	3
Wine	126	52	13	3
Balance	499	126	4	3
Zoo	70	31	16	7
Image segmentation	1617	693	19	7
Ecoli	235	101	7	8
Multiple features	1400	600	649	10
Jaffe	149	64	4096	7

**Table 2:** Results of Experiments on classification problem.

Datasets	Algorithms	Training time (s)	Testing accuracy	Hidden nodes
Ionosphere	BP	33.944864	82.8571	20
	ELM	0.667364	87.6190	40
	TROP-ELM	0.6517	89.2900	51
	Proposed	**0.396109**	**92.3810**	**31**

Iris	BP	29.232330	93.3333	15
	ELM	0.235005	95.5556	40
	TROP-ELM	0.0738	96.6700	59
	Proposed	**0.052971**	**100**	**15**

Wine	BP	31.336370	94.2308	18
	ELM	0.218872	96.1538	35
	TROP-ELM	0.1242	96.5800	84
	Proposed	**0.092291**	**100**	**22**

Balance	BP	136.802525	76.1905	10
	ELM	1.121717	83.0688	28
	TROP-ELM	0.3224	87.2100	56
	Proposed	**0.474296**	**87.5661**	**13**

Zoo	BP	34.132912	80.6452	10
	ELM	0.082092	93.5484	15
	TROP-ELM	0.0316	94.5000	18
	Proposed	**0.030456**	**97.2350**	**7**

Image segmentation	BP	428.141558	87.8582	15
	ELM	24.629949	91.3008	90
	TROP-ELM	207.8026	90.4300	187
	Proposed	**11.964243**	**96.4131**	**70**

Ecoli	BP	55.678908	71.4286	15
	ELM	0.646599	85.9890	40
	TROP-ELM	0.5869	92.0700	90
	Proposed	**0.252657**	**93.6813**	**26**

Multiple features	BP	226.625993	90.9000	12
	ELM	67.085971	97.6833	180
	TROP-ELM	190.431	98.4300	338
	Proposed	**40.753813**	**99.0333**	**121**

Jaffe	BP	427.894980	75.4464	20
	ELM	1.778349	81.9196	130
	TROP-ELM	1.668231	83.4550	240
	Proposed	**1.530555**	**84.3750**	**99**

## Data Availability

The data used to support the findings of this study have been deposited in the UCI Machine Learning Repository and the Psychology Department in Kyushu University.
